# Nuclear magnetic resonance-based metabolomic study of rat serum after anterior cruciate ligament injury

**DOI:** 10.1038/s41598-023-46540-y

**Published:** 2023-11-07

**Authors:** Jie-Ting Li, Ni Zeng, Zhi-Peng Yan, Tao Liao, Xin Chen, Guo-Xin Ni

**Affiliations:** 1 Department of Rehabilitation Medicine, Fuzhou Second Hospital, Fuzhou, China; 2 Department of Rehabilitation Medicine, The Affiliated Hospital of Guizhou Medical University, Guizhou, China; 3 Department of Rehabilitation Medicine, First Affiliated Hospital of Fujian Medical University, Fuzhou, China; 4 Department of Rehabilitation Medicine, Chengdu Second People’s Hospital, Chengdu, China; 5 Department of Rehabilitation Medicine, The First Affiliated Hospital of Xiamen University, Xiamen, China

**Keywords:** Chemical biology, Rheumatology

## Abstract

Anterior cruciate ligament (ACL) injury, a common sports injury, is associated with a high risk of subsequent osteoarthritis (OA), which can cause serious pain and disability. Understanding the detailed mechanism underlying the predisposition of knee with ACL injury to secondary OA at an early stage is key to preventing future degradation and progression to a clinically significant disease. A total of 56 male Sprague Dawley rats (age, 8 weeks; weight, 180–220 g) were randomly divided into three experimental groups: control, ACL transection (ACLT; where surgical procedure was performed with ACLT), and sham (where surgical procedure was performed without ACLT). The ACLT and sham groups were further divided into three subgroups based on when the rats were sacrificed: 4, 8, and 12 weeks after the surgical procedure. The control group and the aforementioned subgroups contained 8 rats each. We used nuclear magnetic resonance (NMR)-based metabolomic analysis to analyze rat serum samples for the metabolic characteristics and the underlying mechanisms. In total, 28 metabolites were identified in the NMR spectra of the rat sera. At 4 and 8 weeks postoperatively, the sham group demonstrated metabolic profiles different from those of the ACLT group. However, this difference was not observed 12 weeks postoperatively. In total, five metabolites (acetate, succinate, sn-glycero-3-phosphocholine, glucose, and phenylalanine) and five metabolic pathways (phenylalanine, tyrosine, and tryptophan biosynthesis; phenylalanine metabolism; pyruvate metabolism; starch and sucrose metabolism; and histidine metabolism) demonstrated significant differences between the ACLT and sham groups. ACL injury was noted to considerably affect biochemical homeostasis and metabolism; however, these metabolic changes persisted briefly. Moreover, glucose was a characteristic metabolite, and several energy-related metabolic pathways were significantly disturbed. Therefore, an ACL injury may lead to considerable impairments in energy metabolism. Abnormal glucose levels facilitate chondrocyte function impairment and thereby lead to OA progression. Furthermore, lactate may aid in identifying metabolic changes specific to knee trauma not related to an ACL injury. Overall, the metabolic changes in rat serum after an ACL injury were closely related to disturbances in energy metabolism and amino acid metabolism. The current results may aid in understanding the pathogenesis of posttraumatic osteoarthritis.

## Introduction

Exercise and sports afford numerous health benefits. However, they also increase injury and illness risks. A major long-term consequence of sports injuries is the risk of posttraumatic osteoarthritis (PTOA) onset at a young age^1^. The prevalence of PTOA after anterior cruciate ligament (ACL) injury, a common sports injury, can be as high as 87%^2^. Even with surgical reconstruction, PTOA incidence remains high^3^. In 50% of the cases, patients may develop osteoarthritis (OA) 5–15 years after the initial ACL injury. Moreover, approximately 50% of patients with an ACL-reconstructed knee may develop OA within 12–14 years of the initial injury^4^. Nevertheless, PTOA etiology and pathogenesis in patients with a history of ACL injury remains unclear. Therefore, the most effective disease-modifying treatment for PTOA remains unavailable.

PTOA commonly has a known “starting point,” which develops after joint injury. Therefore, in theory, if appropriate interventions are initiated at an early stage, PTOA progression may be prevented. Understanding the mechanism underlying PTOA development is a prerequisite for developing a novel diagnostic method for early osteoarthritis detection and for effective PTOA treatment. The pathogenesis of secondary OA after ACL injury may be multifaceted, including pathological mechanical stress, joint inflammation, and metabolic disturbances^5^. An injured ACL cannot maintain the knee joint as stably as an uninjured ACL can. Therefore, in the case of an injured ACL, the mechanical load becomes distributed to other structures, resulting in matrix loss and chondrocyte apoptosis^6^. Moreover, various biological factors, such as cytokines, molecular biomarkers, and oxygen free radicals, may disturb homeostasis and trigger progressive joint degeneration^7^. Furthermore, metabolic disturbances may contribute to PTOA development^8,9^. As a disease of the whole joint organ, OA can induce irreversible metabolic changes in various joint tissues^10^.

Metabolomic techniques reported thus far have been demonstrated to have great potential for exploring various diseases. As such, metabolomic analyses for OA are emerging as a new research hotspot^11^. Metabolomics—a member of the family of the -omics sciences (in addition to genomics, transcriptomics, and proteomics) that is developing rapidly—include untargeted and targeted analyses of metabolites in body fluids or tissues^12^. These metabolites include small molecules that characterize an individual’s phenotype and provide valuable information about them. Therefore, novel metabolic information has the potential to create innovative ideas for clinical diagnosis and treatment interventions. Lupton et al.^13^ demonstrated the application of metabolomics in the context of gliomas and brain tumor biology. Moreover, Tayeb et al.^14^ reported the development of potential metabolomic-related pharmacotherapies for evaluating treatments for psychotic disorders. Moreover, impaired energy metabolism, lipid metabolism, amino acid metabolism, and other metabolic factors may be associated with OA pathogenesis^15^.

Early identification of PTOA (before it reaches an irreversible stage) may facilitate the development and improvement of approaches for identifying early disease processes and tracking treatment responses. However, metabolomic studies for continuous and dynamic observation at different stages of OA onset and progression have not been reported thus far. In the literature, a large amount of evidence indicates that lesions in the ACLT rat progressed from mild-to-moderate OA during 4–8 weeks, to moderate-to-severe OA at 12 weeks post-surgery. At later phases (8–12 weeks), the irreversible destruction of articular cartilage and subchondral bone is characteristic in the late stage of OA^16–18^. We therefore performed NMR-based metabolomic analysis to dynamically analyze the metabolic characteristics and related mechanisms in rat models with ACL transection (ACLT) after 4, 8, and 12 weeks postoperatively. We aimed to mechanistically understand changing metabolic profiles, identify major metabolites with markedly altered levels and significantly disturbed metabolic pathways, and identify potential biomarkers of a knee joint with an ACL injury. The current results may aid in understanding the metabolic alterations underlying ACL injury progression, as well as the mechanisms underlying PTOA pathogenesis.

## Materials and methods

### OA animal model

This study was approved by the Animal Ethics Committee of Fujian Medical University. We confirm that all experiments were performed in accordance with relevant guidelines and regulations. Anesthesia was induced through isoflurane inhalation, with minimal distress caused to the animals. The rats were housed in plastic cages under controlled environmental conditions with ad libitum access to standard rodent chow and water.

In total, 56 male Sprague Dawley rats, aged 8 weeks and weighing 180–220 g, were randomly allocated to one of these three experimental groups: control, ACLT, and sham (where the surgical procedure was performed without ACLT). Both the ACLT and sham groups were divided into three subgroups each on the basis of the timepoints at which the rats were euthanized: 4, 8, and 12 weeks postoperatively. The control group and all the aforementioned subgroups contained 8 rats each.

**Figure 1:**
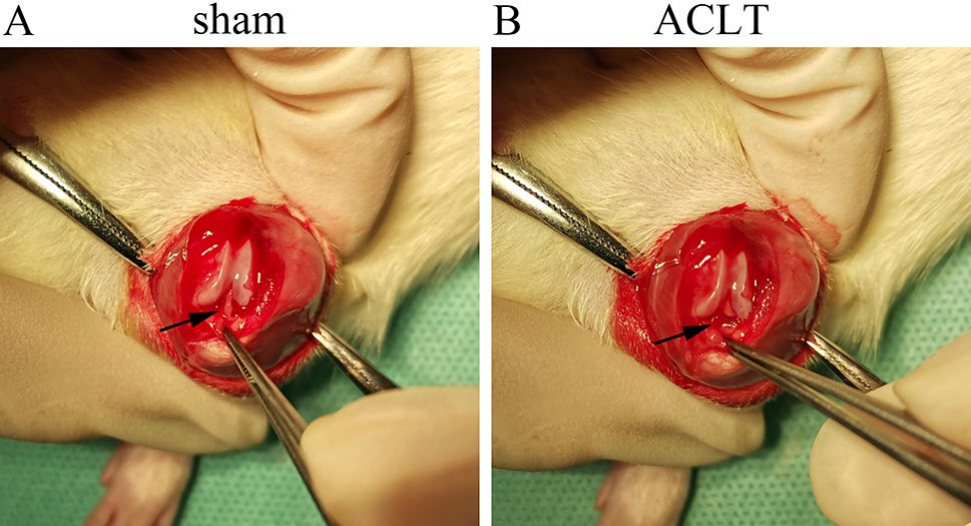
Post-traumatic osteoarthritis mouse model. (**A**) sham group, the black arrow is the anterior cruciate ligament. (**B**) ACLT group, the black arrow is the severed nerves ACL.

In each rat, OA was induced through surgical transection of the left ACL. In brief, anesthesia was induced through 2%–3% isoflurane inhalation for induction and 1%–2% for maintenance. The anesthesia time for rats is 4–5 min. Inhalation of isoflurane was diminished and stopped postoperatively. The left knee was shaved and prepared using an iodine solution. Next, a 2-cm-long median incision was made at the knee joint. Thereafter, the subcutaneous tissue and muscle were incised, and a longitudinal incision was made along the medial side of the white patellar ligament. The joint capsule was then opened with the limb hyperextended, and then the patella was turned to the outside. With the limb in full flexion, the ACL was visualized through blunt dissection and sectioned using a scalpel blade. Next, the patella was reset, and the knee joint was straightened. Successful ACLT was confirmed using the anterior drawer test. In this test, the rat lies supine with knees flexed to 90 degrees to relax the knee muscles. We fix paw, grasps the tibia of the surgical limb just below the knee, and draws the tibia forward. The significant laxity caused by the tibial plateau sliding anteriorly compared with that of the contralateral knees was considered positive**.** For hemorrhage and hematoncus prevention, the joint capsule was washed with sterile saline solution. The joint capsule and muscle layers were then closed. For sham surgery, we performed all the aforementioned steps except ACLT. The mean operating time for the ACL group was 30 min and for the sham group was 15 min approximately. In Fig. 1, black arrows indicate the ACL and severed nerves of ACL. The rats were administered penicillin (400,000 IU, intramuscularly) for 3 days postoperatively to prevent infection.

Rats from all groups were allowed to move freely in their plastic cages without any fixing measures for 4, 8, or 12 weeks postoperatively. Over the course of the experiment, we observed the general condition of the rats and recorded their weights every week. At the sampling timepoint, we euthanized the rats and collected their blood through cardiac puncture. The rats were maintained in abrosia for 12 h before the euthanasia. All the blood samples were kept at room temperature for 1–2 h and then centrifuged at 2,000 × g for 20 min. The supernatant was aspirated into another tube and stored at − 80 °C until the nuclear magnetic resonance (NMR)-based metabolomic analysis.

### NMR-based metabolomic analysis and NMR data analysis

Before the analyses, the frozen serum samples were thawed on ice, and 0.1 M phosphate buffer (pH 7.4) was prepared by dissolving 50 mM K_2_HPO_4_/NaH_2_PO_4_ in 20% D_2_O. Next, 250 µL of a thawed serum sample was mixed with 250 µL of the 0.1 M phosphate buffer (pH 7.4) in an Eppendorf tube, followed by centrifugation at 12,000 × g at 4 °C for 10 min. Next, 500 µL of the supernatant (almost 100% of sample) was transferred into a 5-mm-diameter NMR tube, followed by low-speed (1,000 r/min) centrifugation at room temperature for 5 min to remove the solution on the pipe wall to the bottom of the centrifuge tube for further metabolomics analysis. This protocol was based on the previously described method^19^.

All 1H-NMR experiments were performed on a Bruker Avance III 850 MHz spectrometer (Bruker BioSpin, Ettlingen, Germany) equipped with a TCI cryoprobe at 25 °C. The results were acquired using the Carr–Purcell–Meiboom–Gill pulse sequence with water suppression: RD-90-(τ-180-τ)n-ACQ, where RD denotes the relaxation delay and ACQ the acquisition time. In total, 32 transients were collected into 64 K data points by using a spectral width of 20 ppm as well as an RD and ACQ of 4 and 1.93 s, respectively. Moreover, pulsed gradients G1 and G2 were used to improve water suppression quality. For preliminary identification of all the metabolites, the peaks in the 1-dimensional (1D) 1H-NMR spectra were initially fitted using Chenomx NMR Suite (version 8.3; Chenomx Inc., Canada). Additionally, the identification process was aided by referring to the Human Metabolome Database (HMDB; http://www.hmdb.ca/) and relevant studies^20^. Two-dimensional (2D) NMR spectroscopy can be useful for increasing signal dispersion and for elucidating the connectivities between signals, thereby helping to identify biochemical substances. To further match chemical shifts and confirm the identification results, 2D 1H-13C heteronuclear single quantum coherence (HSQC) spectra and 2D 1H-1H total correlation spectroscopy (TOCSY) spectra were utilized.

Phase correction, baseline correction, and resonance alignment were performed for all 1D 1H NMR spectra using MestReNova (version 9.0.1). The NMR spectra were obtained with the methyl group of lactates (*δ* 1.33) as the reference. The region of water resonance (*δ* 4.70–5.00) was removed from the spectra to eliminate baseline distortion due to imperfect water saturation. The 1D 1H spectral region (*δ* 0.7–4.7 and 5.0–9.0 ppm) was segmented into 0.002-ppm-wide bins for further statistical analysis. The relative integral value of the independent spectral peak was selected as the relative concentration of the corresponding metabolite. Here, the relative concentrations of the metabolites are expressed as means ± standard deviations.

We next performed multivariate data analysis using SIMCA-P + 14.0; we scaled the data through Pareto scaling to increase the importance of low-level metabolites without significant noise amplification. Unsupervised principal component analysis (PCA) approximates the variation by a low-dimensional model plane to observe the grouping trends and show clusters among the groups. Supervised partial least-squares discriminant analysis (PLS-DA) was subsequently performed to check grouping trends, improve group separation, and extract the correlated variables related to sample belongings. Permutation tests were performed with 200 iterations to validate the PLS-DA model for robustness. The extracted R2 and Q2 values reflected the explained variance and predictive capabilities. The reliability of the model was increased with R2 and Q2 approaching to 1. On the basis of the validated PLS-DA models, the metabolites with the variable importance in projection (VIP) of > 1 were identified to be significant metabolites.

Univariate analysis was conducted using MATLAB Statistics Toolbox in SPSS (version 19.0; SPSS, Chicago, IL, USA). Student’s *t*-test was used for evaluating the significance of the mean values between the two groups. We also performed one-way analysis of variance (ANOVA) followed by Bonferroni multiple comparison test and identified metabolites with a *p*-value of < 0.05 as differential. Characteristic metabolites were identified by integrating significant and differential metabolites.

A significantly altered metabolic pathway analysis was performed based on the identified characteristic metabolites by using Kyoto Encyclopedia of Genes and Genomes (KEGG) database data (http://www.kegg.jp/kegg/pathway.html) and MetaboAnalyst (version 4.0; https://www.metaboanalyst.ca). The metabolic pathways with *p* < 0.05 and pathway impact value (PIV) > 0.2 were identified to be significantly disturbed metabolic pathways.

### Ethics approval and consent to participate

The animal study was reviewed and approved by the Animal Ethics Committee of Fujian Medical University.

## Results

### PTOA-related anatomical and physiological changes in rats

In this experiment, we used the PTOA rat models to explore the metabolic changes caused by a knee ACL injury. All surgical procedures were successful without any adverse incidents related to anesthesia or death. Postoperative recovery, including the healing of the surgical incision, was also uneventful, with no infection or death. The mean weight of the control rats (*n* = 8) was 204.3 ± 9.0 g. As shown in Fig. S1, before the experiment, the between-group rat weight differences were nonsignificant (*p* > 0.05). Over the entire experimental period, the sham and ACLT group rats’ weight, measured weekly, demonstrated a steady upward trend, but the weight gain in the ACLT group was slower. Nevertheless, no other difference was noted between the sham and ACLT groups at any timepoint (*p* > 0.05).

### Metabolic profile analysis of rat sera

Fig. S2 illustratesthe typical 1H-NMR spectra of sera from the two groups. In total, 28 metabolites were identified in the NMR spectrum (Table S1), all of which were confirmed using the 2D 1H-13C HSQC and 1H-1H TOCSY spectra (Figs. S3 and S4).

**Figure 2:**
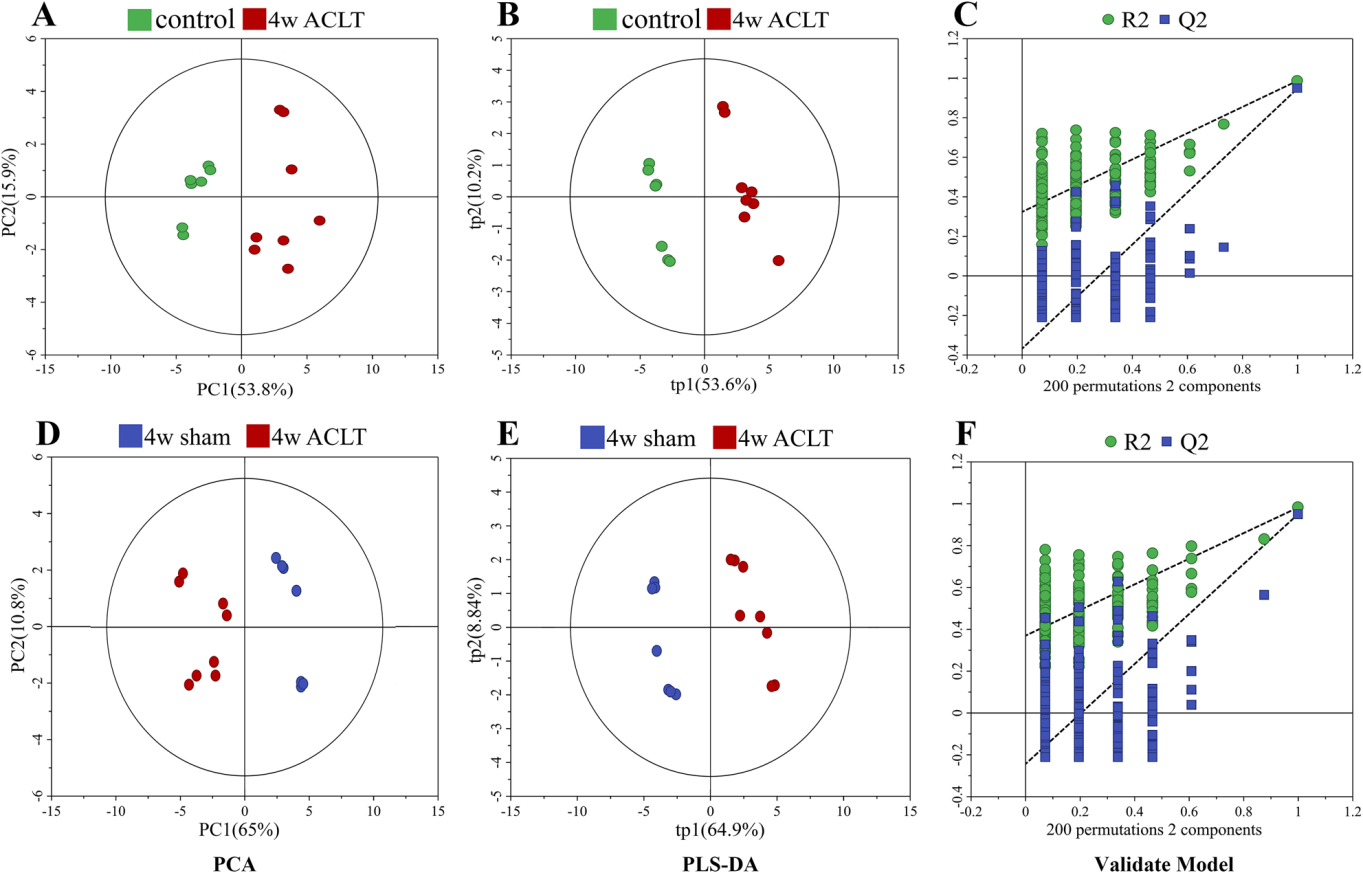
Multivariate statistical analysis chart in the 4-week after the operation. Pair-wise PCA scores plots of the control group vs. 4-week ACLT group (**A**), 4-week sham group vs. 4-week ACLT group (**D**). The scores plots of PLS − DA models (**B**, **E**) and validations for pair-wise comparisons (**C**, **F**) of these groups.

**Figure 3:**
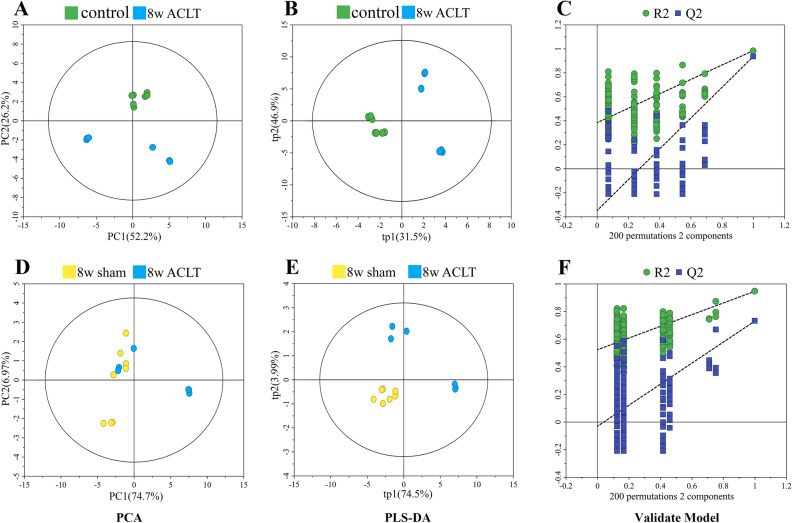
Multivariate statistical analysis chart in the 8-week after the operation. Pair-wise PCA scores plots of the control group vs. 8-week ACLT group (**A**), 8-week sham group vs. 8-week ACLT group (**D**). The scores plots of PLS − DA models (**B**, **E**) and validations for pair-wise comparisons (**C**, **F**) of these groups.

**Figure 4:**
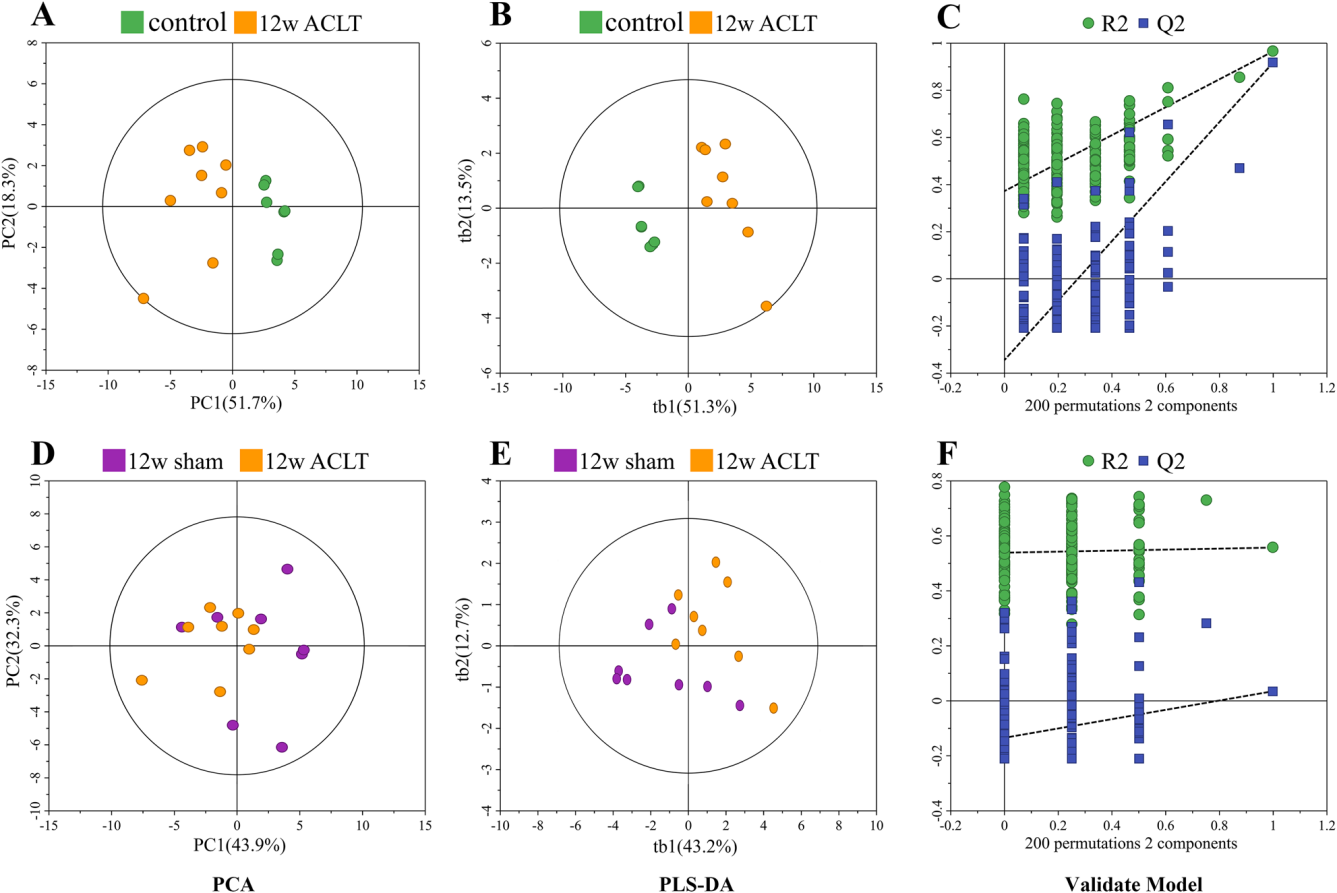
Multivariate statistical analysis chart in the 12-week after the operation. Pair-wise PCA scores plots of the control group vs. 12-week ACLT group (**A**), 12-week sham group vs. 12-week ACLT group (**D**). The scores plots of PLS − DA models (**B**, **E**) and validations for pair-wise comparisons (**C**, **F**) of these groups.

For comprehensive observation of the metabolic profiles from the sham and ACLT group rats at 4, 8, and 12 weeks postoperatively, we performed unsupervised PCA on the relevant NMR data. The results are shown in Figs. 2, 3 and 4, where the dot represents the metabolic modes of the samples. Next, we assessed the metabolic distinctions using untargeted PLS-DA, with response permutation tests with 200 cycles to confirm the PLS-DA models’ reliability.

In the PCA scatter plot at 4 weeks postoperatively, the metabolic profiles of the sham group could be somewhat differentiated from those of the ACLT group. In the PCA scatter plot at 8 weeks postoperatively, this clear separation became clearer. However, in the PCA scatter plot at 12 weeks postoperatively, the dots for the sham and ACLT group rats were mixed with each other, without any separation; model validation also failed for this timepoint. This result suggested that the metabolic patterns of the sham and ACLT group rats did not demonstrate any differences 12 weeks postoperatively. Nevertheless, at all three timepoints, the control group exhibited metabolic profiles that were distinct from those of the ACLT group.

## Statistical analysis results

**Figure 5:**
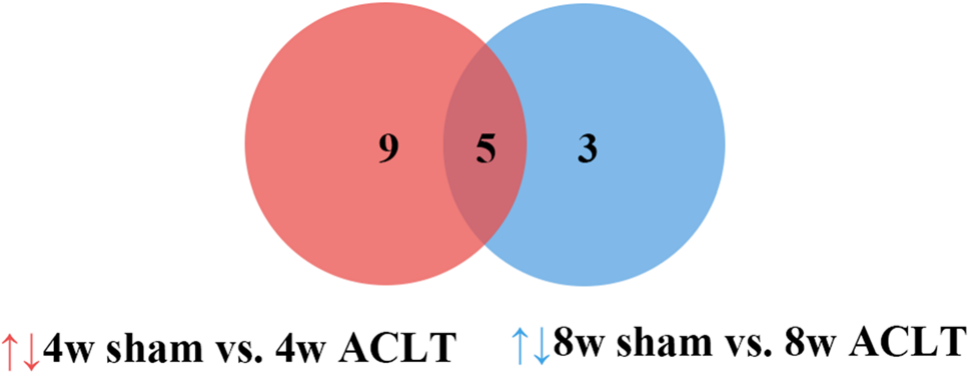
Wayne diagram of characteristic metabolites. 5 metabolites were picked out as potentially important variables for the separation between OA samples in the 4-week and 8-week ACLT group compared with the sham group: acetate, succinate, sn-glycero-3-phosphocholine, glucose and phenylalanine.

Significant and differential metabolites were integrated to identify characteristic metabolites. One-way ANOVA was used to compare the levels of differential metabolites based on relative integrals, which were calculated from the 1D 1H-NMR spectra of the serum samples shown in Tables S2–S4. VIP score-ranking plots of significant metabolites were identified from the PLS-DA models shown in Fig. S5. At 4 and 8 weeks postoperatively, the sham and ACLT groups demonstrated 14 and 8 characteristic metabolites (Table 1). Moreover, seven, eight, and nine characteristic metabolites were shared between the ACLT and control groups at 4, 8, and 12 weeks postoperatively, respectively. As shown in Fig. 5, the five identical characteristic metabolites shared by the sham and ACLT groups 4 and 8 weeks after the operation were acetate, succinate, sn-glycero-3-phosphocholine, glucose, and phenylalanine; all five of these were considered potentially important variables for separating OA samples.

**Table 1:** Characteristic metabolites identified in different postoperative stages of rat serum.

Metabolite	4w sham vs. 4w ACLT P change	8w sham vs. 8w ACLT P change	Control vs. 4w ACLT P change	Control vs. 8w ACLT P change	Control vs. 12w ACLT P change
Valine				** ↑	** ↑
Isoleucine	*** ↓			** ↑	* ↑
LDL/VLDL		** ↓	** ↓		
3-HB *	*** ↓				
Lactate	*** ↓		** ↑		
Lysine	*** ↓			*** ↑	* ↑
Acetate	*** ↓	* ↑			*** ↓
Arginine				** ↑	
Pyruvate	*** ↓		** ↑		
Succinate	*** ↓	* ↑			
Citrate	*** ↑				
Creatine			*** ↑		** ↑
Choline				** ↑	** ↑
PC*	*** ↓				
GPC*	** ↑	*** ↓	** ↓	*** ↓	*** ↓
Taurine	*** ↑		*** ↓	*** ↓	*** ↓
Glycerol					
Glucose	*** ↑	** ↑	*** ↓		
Fumarate		* ↑			
Tyrosine		* ↑			
Phenylalanine	*** ↓	*** ↑			*** ↑
Formate	*** ↓			*** ↓	

*p < 0.05, **p < 0.01, ***p < 0.001.*: 3-HB, 3-Hydroxybutyrate; PC, O-phosphocholine; GPC, sn-Glycero-3-phosphocholine.

### Metabolic pathway analysis results

**Figure 6:**
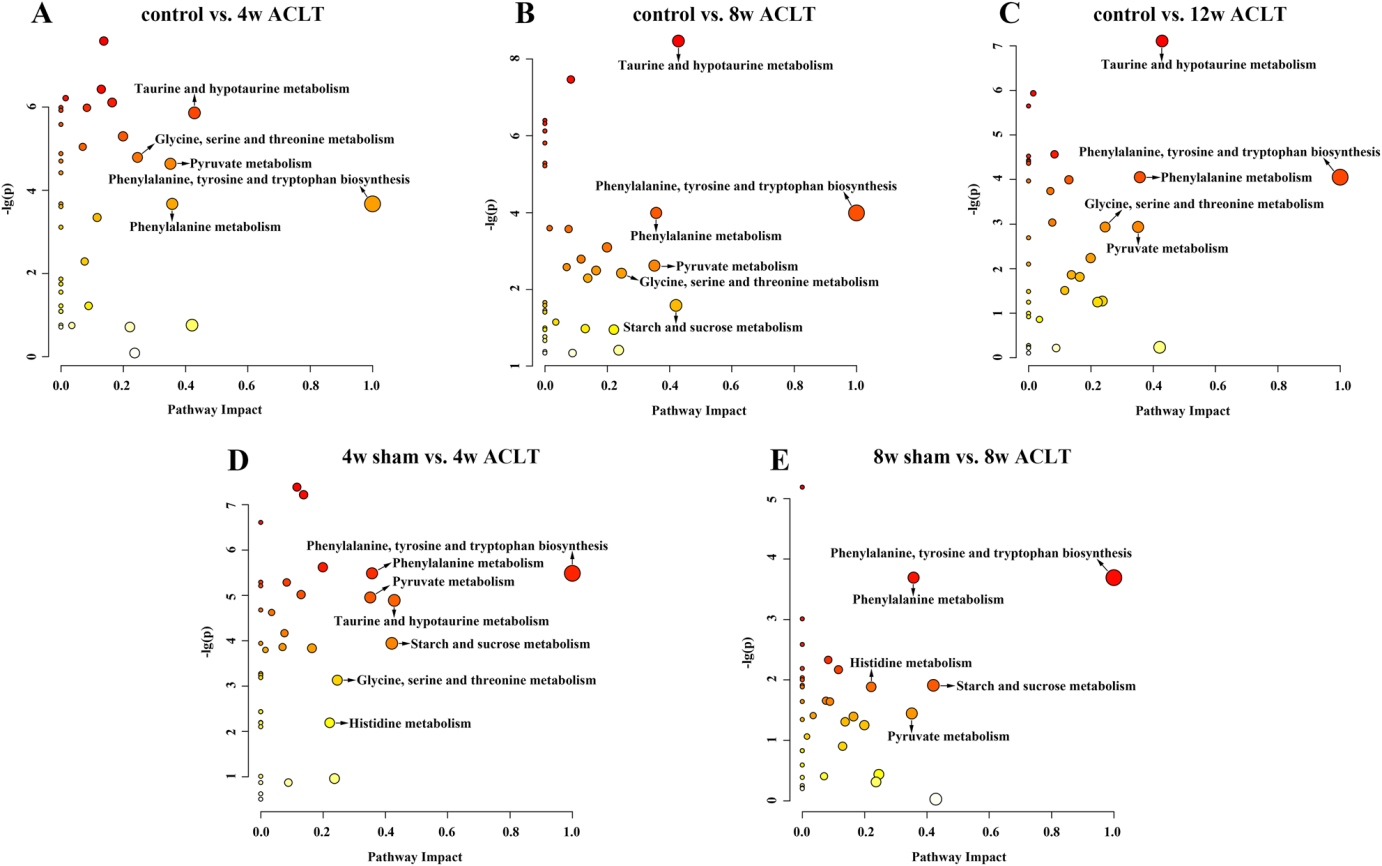
Metabolic pathway analysis. (**A**) control group vs. 4-week ACLT group; (**B**) control group vs. 8-week ACLT group; (**C**) control group vs. 12-week ACLT group; (**D**) 4-week sham group vs. 4-week ACLT group; (**E**) 8-week sham group vs. 8-week ACLT group. A bubble represents an identified metabolic pathway. The bubble size is proportional to the pathway impact value (PIV), with the color denoting the statistical significance *p* from highest (in red) to lowest (in white). Metabolic pathways with p value < 0.05 and PIV > 0.05 were identified to be significantly altered metabolic pathways.

**Figure 7:**
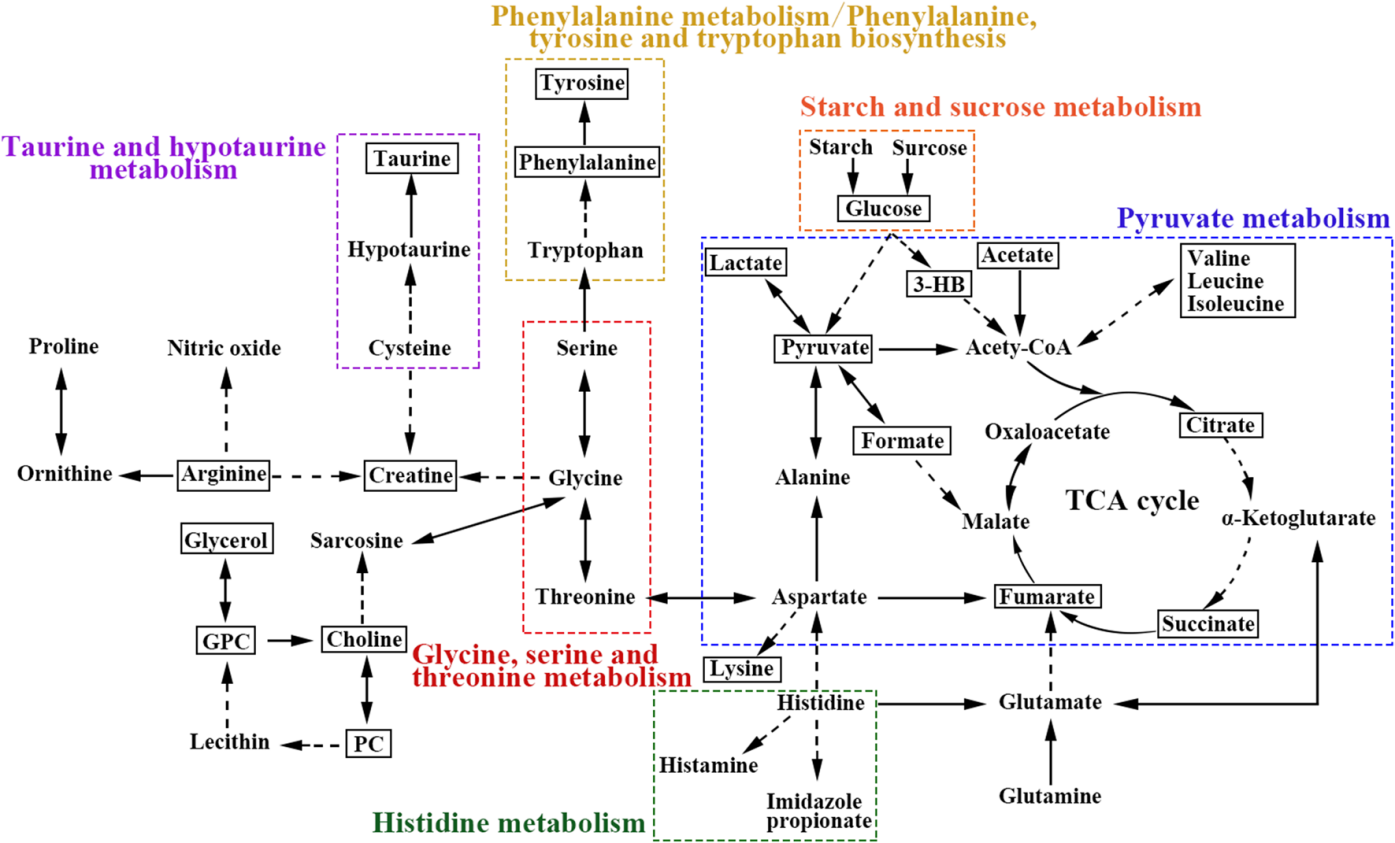
Overview of significantly altered signaling pathways and metabolic pathways.

In Fig. 6, the sizes and colors of the circles are based on the PIVs and *p*-values in the pathway enrichment analysis, respectively. In particular, PIV > 0.2 and *p* < 0.05 were considered to indicate significantly altered metabolic pathways. Table S5 lists the potential significantly altered metabolic pathways at different timepoints postoperatively. Compared with those in the sham group, the ACLT group demonstrated significant alterations 4 and 8 weeks postoperatively in seven and five metabolic pathways, respectively. Similarly, at 4, 8, and 12 weeks postoperatively, the ACLT group demonstrated significant alterations in five, six, and five metabolic pathways compared with those in the control group. In general, at both 4 and 8 weeks postoperatively, these five metabolic pathways consistently appeared to be most relevant to PTOA development: phenylalanine, tyrosine, and tryptophan biosynthesis; phenylalanine metabolism; pyruvate metabolism; starch and sucrose metabolism; and histidine metabolism. By using the KEGG database data, we prepared a schematic of our metabolic pathway analysis (Fig. 7) to intuitively observe the relationship between the characteristic metabolites and the significantly altered metabolic pathways.

## Discussion

The ACLT rat model is excellent for mimicking the changes occurring in a human joint after an ACL injury^21^. In the current study, we systematically reviewed metabolic changes in rats without ACLT (control and sham-operated) and those with ACL injury. The synovial fluid (SF) may be an ideal choice for the study of OA because it is in direct contact with the ACL^22^. However, extracting the SF from small animals or patients, particularly healthy controls, is difficult and requires invasive, destructive surgery. Nevertheless, the metabolic components in the serum may indirectly reflect the biological processes occurring in the knee joint. Zhang et al.^23^ indicated a strong correlation between the plasma and SF in patients with OA. Furthermore, Zhai et al.^24^ reported that serum NMR spectra can be used to discriminate OA cases from the controls and that the metabolic profiles identified from the serum and SF samples were largely identical to each other. These results confirm the potential value of the NMR-based metabolomic study of rat serum after an ACL injury.

In the current study, multivariate analysis revealed that the serum metabolic profiles changed significantly over 4 and 8 weeks postoperatively in the ACLT group compared with those in the sham group. However, these changes were not apparent 12 weeks postoperatively, suggesting that the differences in the serum metabolic profiles of the ACLT and sham groups diminished by this timepoint. In other words, an ACL injury may affect biochemical homeostasis and metabolism considerably but only over a short term. Our metabolomic study results appear to contradict the observation that the degeneration degree and pathology in a knee with an ACL injury changes over time. Maher et al.^25^ also noted no metabolic differences 12 weeks postoperatively in the PCA models of the NMR spectra of serum samples from their meniscal destabilization (MD), ACLT, and sham groups. Heard et al.^26^ also observed some changes consistent with early OA 20 weeks postoperatively in addition to some significant alterations 2 weeks postoperatively. Here, we used consecutive postsurgical timepoints (i.e., 4, 8, and 12 weeks postoperatively) for our metabolomic analysis; as such, our results accurately demonstrated distinctly altered metabolic profiles and significantly disturbed metabolic pathways in the ACLT group.

Glucose was selected as a characteristic metabolite in our metabolomic analysis because we noted that several energy-related metabolic pathways were significantly disturbed; these pathways included pyruvate metabolism; glycine, serine, and threonine metabolism; and starch and sucrose metabolism. Furthermore, the ACLT group demonstrated alterations in glycolysis and tricarboxylic acid cycle substrates. Therefore, energy metabolism may be significantly impaired after ACLT. Nevertheless, the biochemical processes underlying energy utilization might be reflected by the significant changes in glucose. Epidemiological and clinical studies have demonstrated the coexistence of OA and altered glucose metabolism—such as that in obesity, hypertension, and type 2 diabetes cases^27^. Altered glucose metabolism is significantly related to both the occurrence and progression of OA^28^. Beata et al.^29^ indicated that the high energy demands of the SF in sheep undergoing ACL reconstruction might be reflected by the significant changes in their glucose levels. Chondrocytes—the only cell type present in articular cartilage—are influenced by glucose levels. Higher glucose levels can lead to impairment in chondrocyte function, facilitating OA progression^30^. Under physiological conditions, glucose transporter (GLUT) is responsible for glucose transport and thus the maintenance of serum glucose levels within the normal range^31^. However, impaired GLUT may increase intracellular concentrations of glucose; in particular, this can lead to glucose and reactive oxygen species (ROS) accumulation in the chondrocytes of patients with OA^32^. In addition to cytokines and mechanical stimuli, ROS is a major factor in OA initiation and progression^33^. ROS may, through oxidative modifications, directly regulate transcription factors, including nuclear factor κB, activating protein 1, and hypoxia inducible factor (HIF)-1, eventually leading to protein degradation^34^. Therefore, studies exploring whether antioxidant therapy can facilitate cartilage homeostasis maintenance and prevent its degradation are warranted. Moreover, increased glucose levels can mediate dehydroascorbate (DHA) concentrations within chondrocytes. DHA is essential for collagen synthesis, disturbance in which can further compromise OA cartilage integrity^35^. The cartilage matrix is composed of a collagen network, and type II collagen is the main collagen in the articular cartilage matrix. In the human intervertebral disc (IVD), the mature central IVD cells typically have the phenotype of chondrocytes. Johnson et al.^36^ indicated that glucose inhibits type II collagen synthesis and impairs IVD cell function.

Our rats’ metabolite profiles also indicated an alteration in histidine metabolism. In their metabolomic study, Koh and Backhed^37^ reported that imidazole propionate is present at higher concentrations in the plasma of patients with diabetes; the authors also hypothesized that imidazole propionate impairs insulin signaling by impairing the mechanistic target of rapamycin complex 1 (i.e., mTORC1). However, in the current study, we could not identify imidazole propionate in our 1H NMR spectra. Nevertheless, the aforementioned hypothesis may be confirmed using NMR in combination with other metabolomic methods such as liquid chromatography–mass spectrometry (MS) or gas chromatography–MS.

It was widely recognized that trauma and surgery alone may influence metabolism^25,38^. Here, we identified a metabolic response to knee surgery, which was independent of ACLT or OA, in the rats. Because a study’s definition of the control group can affect the interpretation of its results, we included two control groups in this study: control and sham. The ACLT group demonstrated an opposite trend compared with the control and sham groups for lactate. Thus, lactate may be significantly associated with stress responses. Moreover, lactate may be associated with mortality in severely injured patients with blunt trauma^39^ and thus may have a prognostic value during trauma surgery^38^. Furthermore, Maher et al. suggested a relationship of acetate and trimethylamine N-oxide levels with surgical trauma in sheep^25^. The findings of our study may help us to identify reactive metabolites by the sham operation itself.

In contrast to that in other metabolomic studies, the metabolic characteristics and related mechanisms after an ACL injury—key to the effective prevention of future degradation and progression to a clinically significant disease—were dynamically analyzed in this study. However, the limitations of our study include the small sample size and short study time. Thus, future studies should confirm our results by using a large number of clinical samples for targeted analysis of the metabolic changes identified here so as to develop effective ACL injury treatment and PTOA prevention methods.

## Conclusion

In summary, ACL injury was noted to considerably affect biochemical homeostasis and metabolism. However, these metabolic changes persisted briefly. The metabolic changes in rat serum after an ACL injury were closely related to disturbances in energy metabolism and amino acid metabolism. 5 metabolites (acetate, succinate, sn-glycero-3-phosphocholine, glucose, and phenylalanine) and 5 metabolic pathways (phenylalanine, tyrosine, and tryptophan biosynthesis; phenylalanine metabolism; pyruvate metabolism; starch and sucrose metabolism; and histidine metabolism) demonstrated significant differences between the ACLT and sham groups. Glucose was selected as a characteristic metabolite, and several energy-related metabolic pathways were significantly disturbed. Therefore, an ACL injury may lead to considerable impairments in energy metabolism. Abnormal glucose levels facilitate chondrocyte function impairment and thereby lead to OA progression. Furthermore, lactate may aid in identifying metabolic changes specific to knee trauma not related to an ACL injury. The current results may aid in understanding the metabolic alterations underlying ACL injury progression, as well as the mechanisms underlying PTOA pathogenesis.

### Supplementary Information

Supplementary Figure S1.

Supplementary Figure S2.

Supplementary Figure S3.

Supplementary Figure S4.

Supplementary Figure S5.

Supplementary Information 6.

## Data Availability

The datasets used and/or analysed during the current study are available from the corresponding author on reasonable request.

## Abbreviations

PTOA: Posttraumatic osteoarthritis
ACL: Anterior cruciate ligament
OA: Osteoarthritis
ACLT: Anterior cruciate ligament transection
NMR: Nuclear magnetic resonance
2D: 2-Dimensional
1D: 1-Dimensional
PCA: Principal component analysis
PLS-DA: Partial least-squares discriminant analysis
VIP: Variable importance in projection
ANOVA: Analysis of variance
KEGG: Kyoto Encyclopedia of Genes and Genomes
PIV: Pathway impact value
SF: Synovial fluid
MD: Meniscal destabilization
GLUT: Glucose transporter
ROS: Reactive oxygen species
HIF: Hypoxia inducible factor
DHA: Dehydroascorbate
IVD: Intervertebral disc
MS: Mass spectrometry
