# Supplementary figures and images for: Nuclear magnetic resonance-based metabolomic study of rat serum after anterior cruciate ligament injury
Source: Sci Rep. 2023 Nov 7;13:19321. doi: 10.1038/s41598-023-46540-y (PMC10630467; doi:10.1038/s41598-023-46540-y)

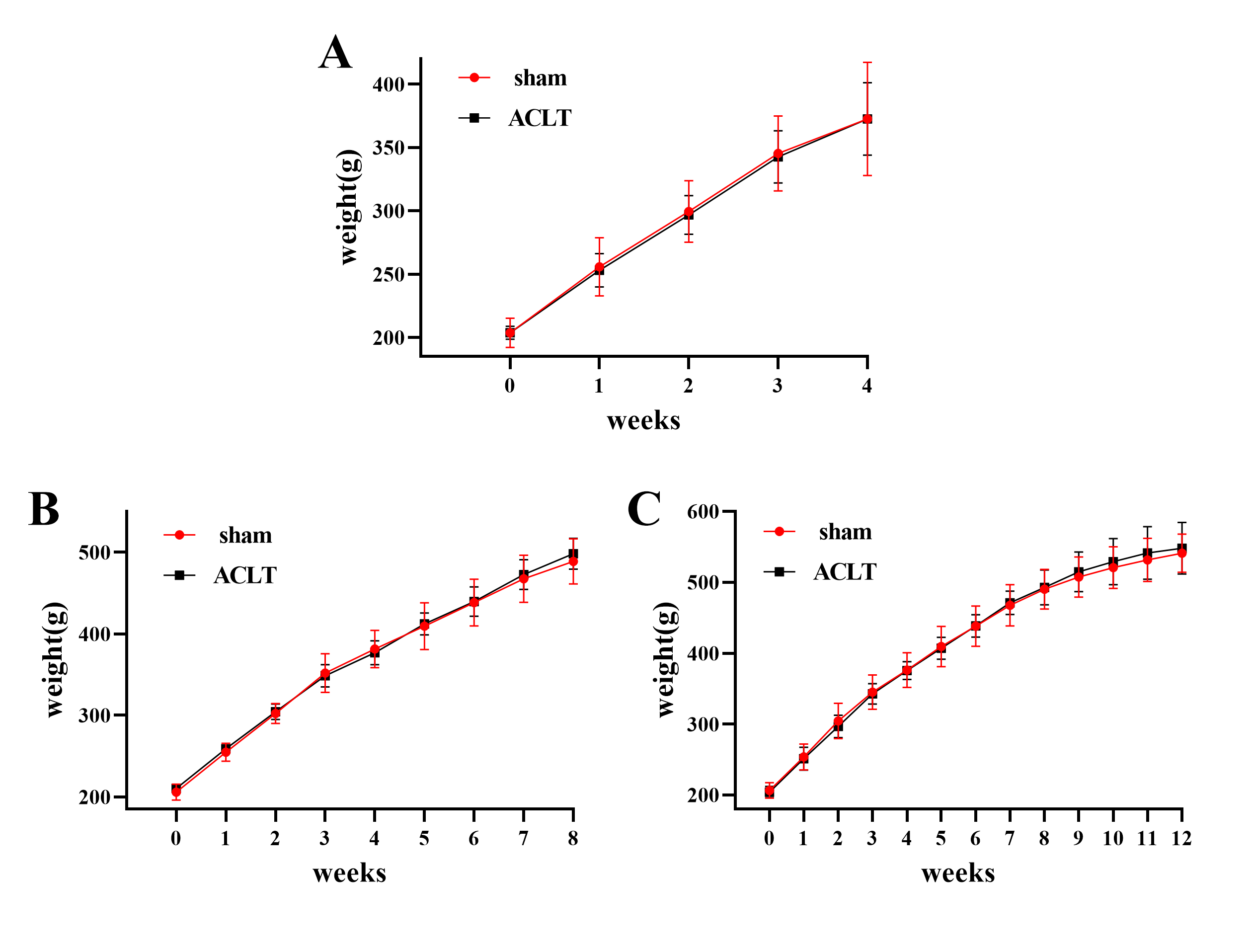

Supplement: Supplementary file 1 — Supplementary Figure S1. [file 41598_2023_46540_MOESM1_ESM.tif]

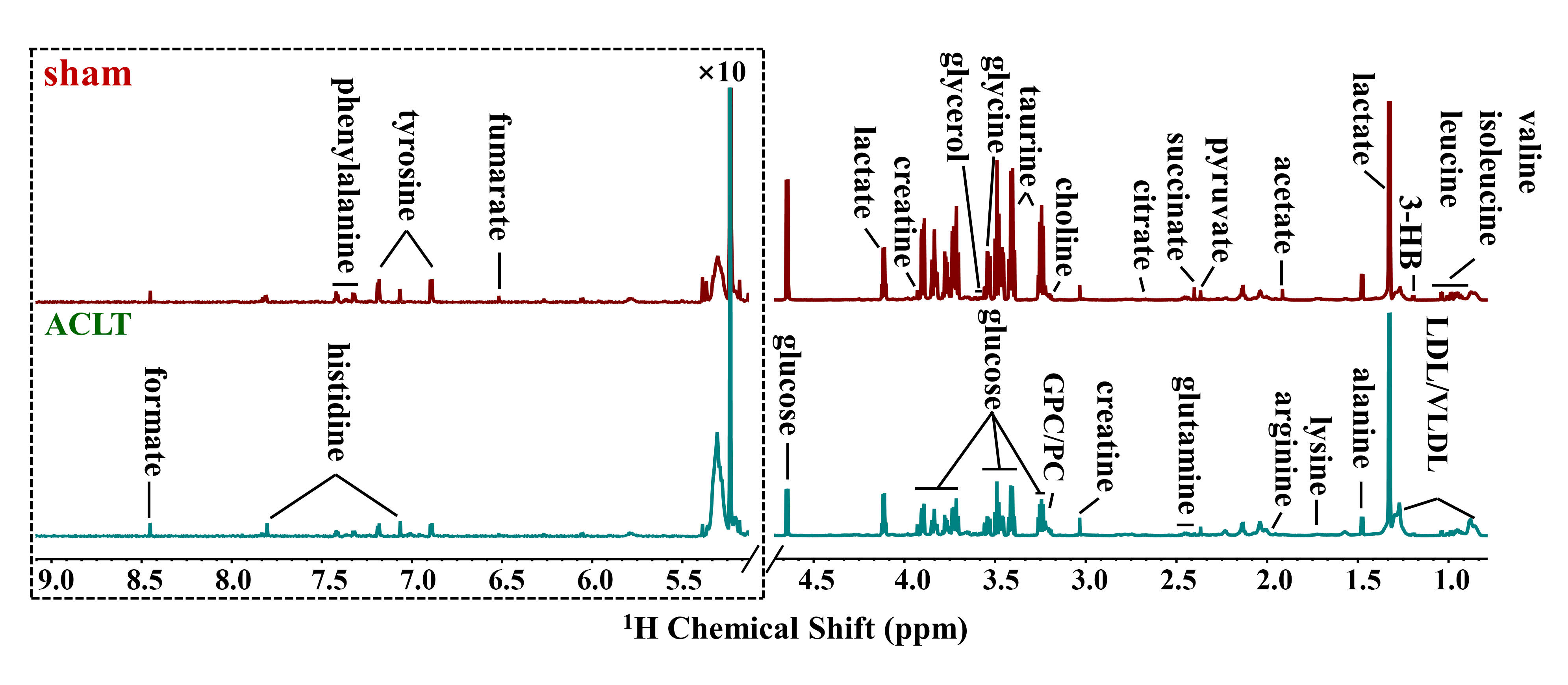

Supplement: Supplementary file 2 — Supplementary Figure S2. [file 41598_2023_46540_MOESM2_ESM.tif]

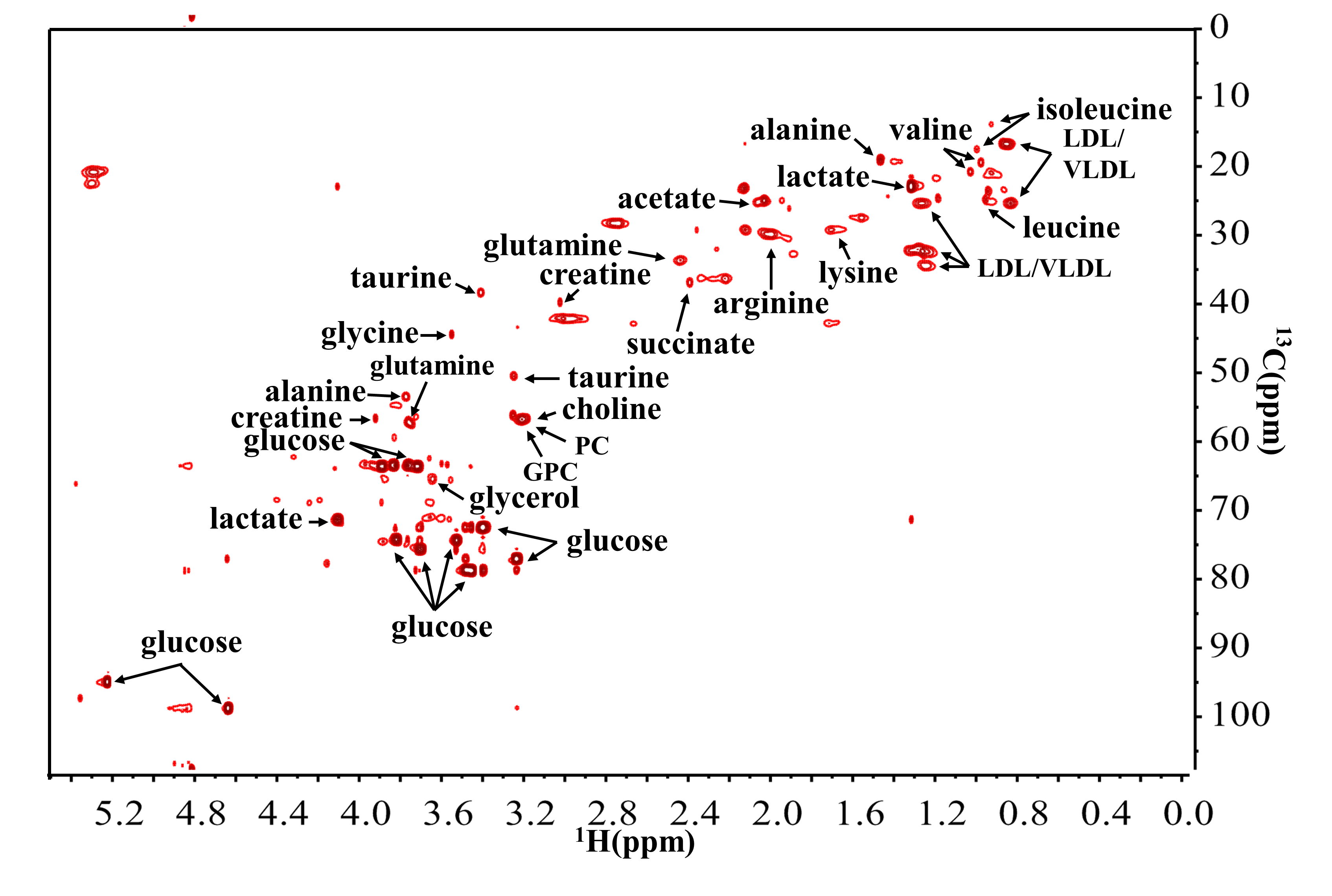

Supplement: Supplementary file 3 — Supplementary Figure S3. [file 41598_2023_46540_MOESM3_ESM.tif]

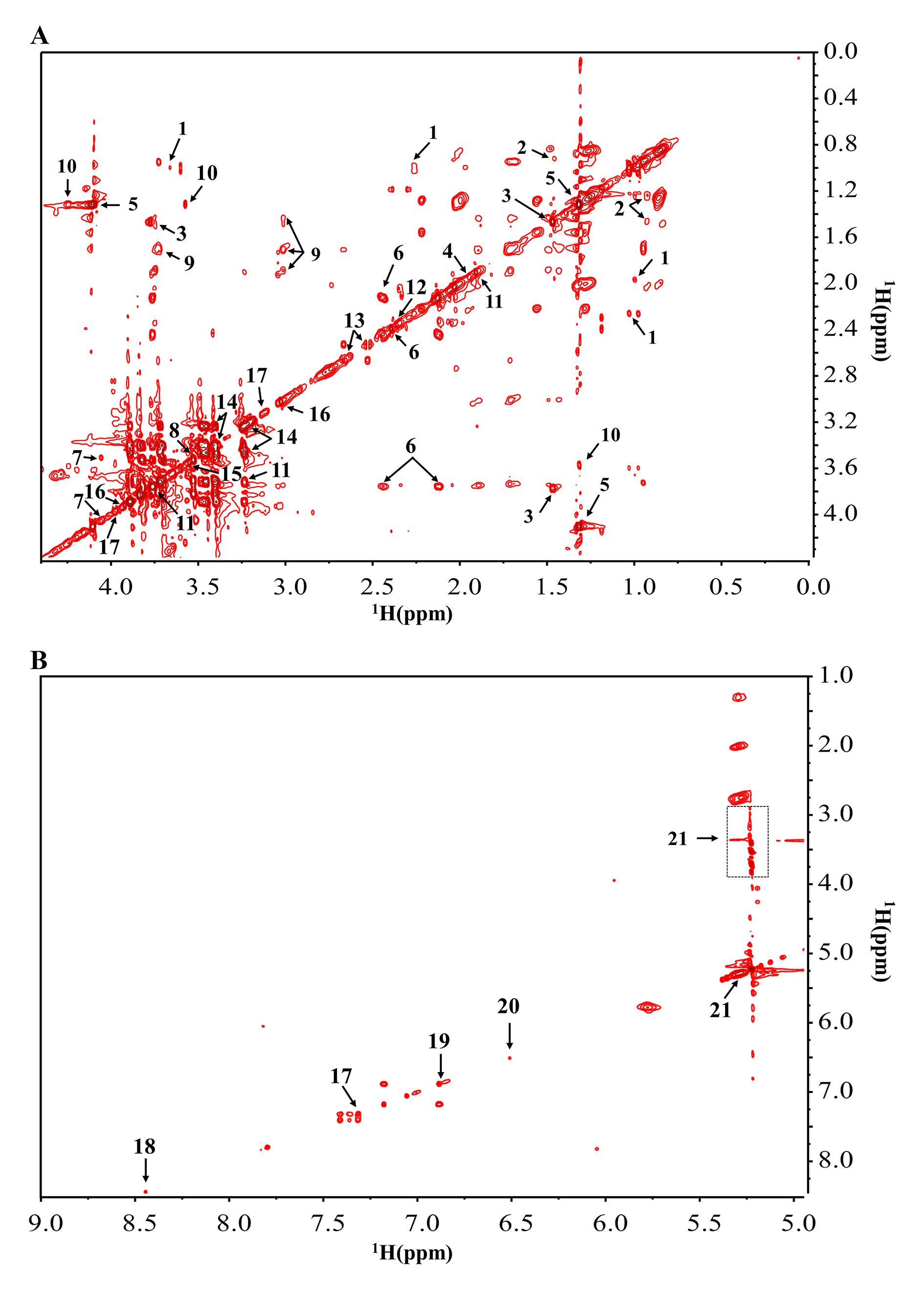

Supplement: Supplementary file 4 — Supplementary Figure S4. [file 41598_2023_46540_MOESM4_ESM.tif]

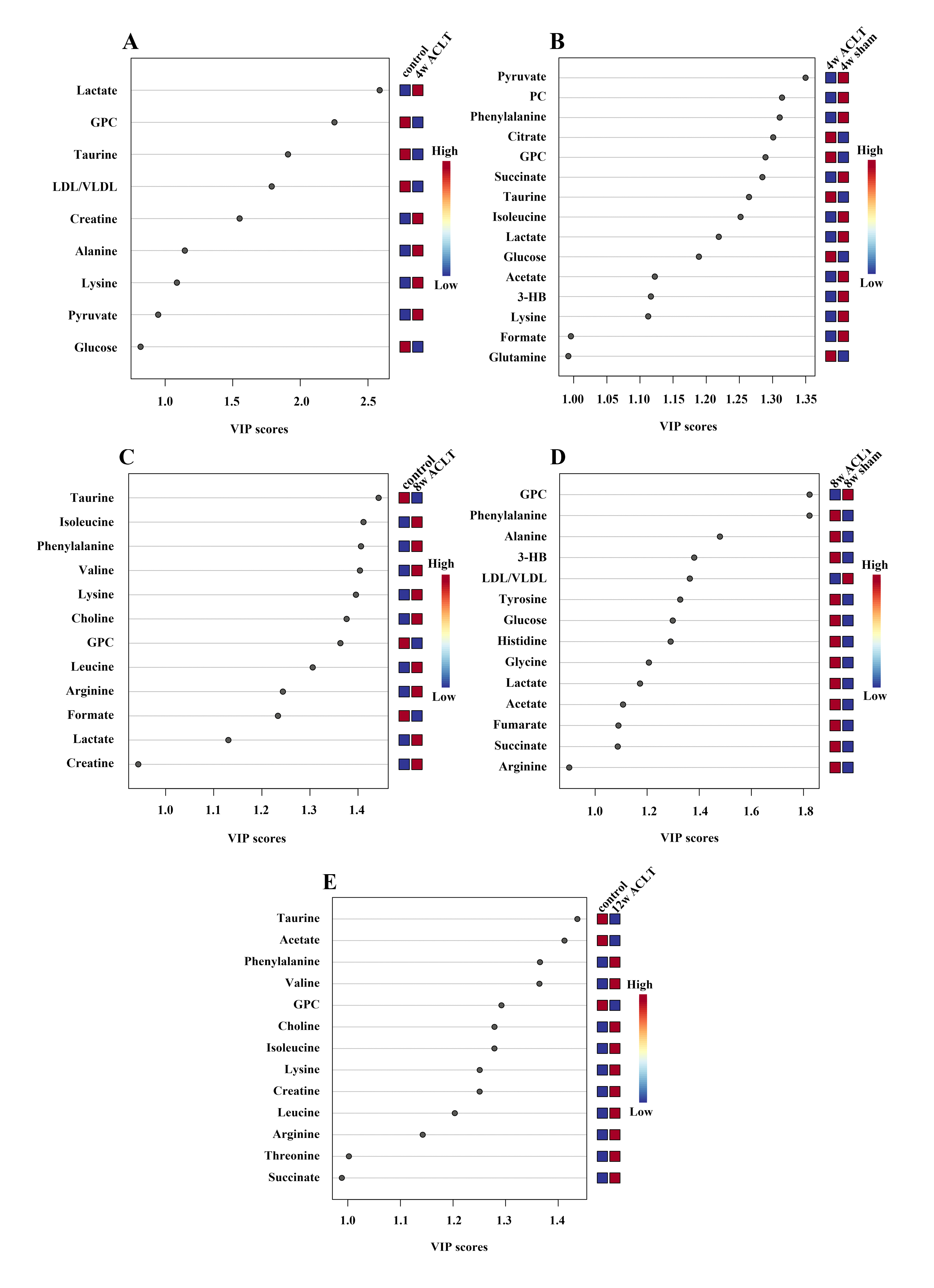

Supplement: Supplementary file 5 — Supplementary Figure S5. [file 41598_2023_46540_MOESM5_ESM.tif]
