# Supplementary material for: Nuclear magnetic resonance-based metabolomic study of rat serum after anterior cruciate ligament injury
Source: Sci Rep. 2023 Nov 7;13:19321. doi: 10.1038/s41598-023-46540-y (PMC10630467; doi:10.1038/s41598-023-46540-y)
Supplement: Supplementary file 6 — Supplementary Information 6. [file 41598_2023_46540_MOESM6_ESM.docx]

**Table S1** Resonance assignments of metabolites in 1H NMR spectra of aqueous extracts derived from rat serum

| **NO.** | **Metabolite** | **δ ^1^H (ppm) and multiplicity#** | **Moieties** |
| --- | --- | --- | --- |
| 1 | Valine | 0.99(d), 1.05(d), 2.26(m), 3.60(d) | γ-CH_3_, γ-CH_3_, β-CH, α-CH |
| 2 | Isoleucine | 0.94(t), 1.01(d), 1.21(m), 1.42(m),  2.00(m), 3.67(d) | δ-CH_3_, γ-CH_3_, half γ-CH_2_,  half γ-CH_2_, β-CH, α-CH |
| 3 | Leucine | 0.96(d), 0.97(d), 1.68(m), 1.71(m),  1.74(m), 3.74(m) | α-CH_3_, α-CH_3_, γ-CH,  β-CH_2_, α-CH |
| 4 | LDL/VLDL | 0.85(t), 0.87(t), 1.25(m), 1.29(m) | CH_3_(CH_2_)n; CH_3_CH_2_CH_2_ =;  CH_2_, (CH_2_)n; CH_2_ CH_2_CO |
| 5 | 3-HB* | 1.20(d), 2.31(m), 2.41(m), 4.16(m) | CH_3_; α-CH; α-CH; CH |
| 6 | Lactate | 1.33(d), 4.11(q) | β-CH_3_, α-CH |
| 7 | Alanine | 1.47(d), 3.78(q) | β-CH_3_, α-CH |
| 8 | Lysine | 1.43(m), 1.49(m), 1.70(m),  1.91(m), 3.02(t), 3.75(t) | half γ-CH_2_, half γ-CH_2_,  α-CH_2_ |
| 9 | Acetate | 1.91(s) | CH_3_ |
| 10 | Arginine | 1.65(m), 1.73(m), 3.23(t), 3.76(t) | γ-CH_2_, δ-CH_3_, α-CH |
| 11 | Pyruvate | 2.37(s) | CH_3_ |
| 12 | Succinate | 2.41(s) | CH |
| 13 | Glutamine | 2.13(m), 2.45(m), 3.77(t) | γ-CH_2_, β-CH_2_, α-CH |
| 14 | Citrate | 2.54(d), 2.65(d) | half-CH_2_, half-CH_2_ |
| 15 | Creatine | 3.04(s), 3.93(s) | N-CH_3_, α-CH_2_ |
| 16 | Choline | 3.21(s), 3.51(dd), 4.04(t) | N-(CH_3_)_3_, N-CH_2_, CH_2_OH |
| 17 | PC* | 3.22(s), 3.60(t), 4.18(m) | N-(CH_3_)_3_, N-CH_2_, CH_2_OH |
| 18 | GPC* | 3.23(s), 3.60(dd), 3.68(dd),  3.87(m), 3.94(m), 4.33(m) | N-(CH_3_)_3_, half ^1^CH_2_, ^2^CH_2_,  half ^1^CH_2_, half ^3^CH_2_,  half ^3^CH_2_, ^1^CH_2_ |
| 19 | Taurine | 3.24(t), 3.41(t) | ^1^CH_2_, ^2^CH_2_ |
| 20 | Glycine | 3.57(s) | α-CH_2_ |
| 21 | Glycerol | 3.55(dd), 3.64(dd), 3.77(m) | half ^1^CH_2_, half ^3^CH_2_, ^2^CH |
| 22 | Threonine | 1.31(d), 3.59(d), 4.25(m) | γ-CH_2_, β-CH |
| 23 | Glucose | β(3.24(dd), 3.48(t), 3.90(dd)),  α(3.54(dd), 3.71(t), 3.72(dd),  3.83(m)) | β(H2, H3, H5),  α(H2, H3, H6) |
| 24 | Fumarate | 6.51(s) | CH |
| 25 | Tyrosine | 3.05(dd), 3.19(dd), 6.92(d), 7.19(d) | half β-CH_2_, half β-CH_2_,  β-CH, α-CH |
| 26 | Phenylalanine | 3.13(dd), 3.32(dd), 3.98(dd),  7.33(d), 7.38(t),7.43(t) | α-CH, half β-CH_2_,  half β-CH_2_, α-CH, β-CH,  γ-CH |
| 27 | Histidine | 7.06(s), 7.85(s) | ^5^CH, ^2^CH |
| 28 | Formate | 8.46(s) | CH |

*: 3-HB, 3-Hydroxybutyrate; PC, O-phosphocholine; GPC, sn-Glycero-3-phosphocholine.

**Table S2** comparison of metabolite levels among the control group, 4-week ACLT group, and 4-week sham group

| **Metabolite** | **Mean ± SD** | | | **Multiple comparisons test** | | **One-way ANOVA** | |
| --- | --- | --- | --- | --- | --- | --- | --- |
|  | **control** | **4w sham** | **4w ACLT** | **control**  **vs.**  **4wACLT** | **4wsham**  **vs.**  **4wACLT** | **F** | **P** |
| Valine | 0.222±0.018 | 0.336±0.039 | 0.296±0.036 | *** | * | 22.722 | <0.001 |
| Isoleucine | 0.146±0.010 | 0.263±0.037 | 0.170±0.017 | NS | *** | 47.578 | <0.001 |
| Leucine | 0.125±0.008 | 0.161±0.020 | 0.136±0.014 | NS | ** | 12.335 | <0.001 |
| LDL/VLDL | 5.751±0.565 | 3.249±0.960 | 4.299±0.664 | ** | * | 20.475 | <0.001 |
| 3-HB * | 0.273±0.063 | 0.335±0.113 | 0.147±0.034 | ** | *** | 12.019 | <0.001 |
| Lactate | 2.758±0.327 | 5.595±0.625 | 3.757±0.681 | ** | *** | 47.457 | <0.001 |
| Alanine | 1.161±0.195 | 1.710±0.276 | 1.396±0.305 | NS | * | 8.095 | 0.003 |
| Lysine | 0.293±0.019 | 0.930±0.290 | 0.474±0.084 | NS | *** | 25.648 | <0.001 |
| Acetate | 0.189±0.022 | 0.238±0.073 | 0.113±0.024 | ** | *** | 14.602 | <0.001 |
| Arginine | 0.045±0.008 | 0.120±0.041 | 0.086±0.020 | ** | * | 14.515 | <0.001 |
| Pyruvate | 0.206±0.029 | 0.738±0.123 | 0.346±0.062 | ** | *** | 84.591 | <0.001 |
| Succinate | 0.040±0.010 | 0.109±0.016 | 0.053±0.018 | NS | *** | 44.781 | <0.001 |
| Glutamine | 0.533±0.111 | 0.385±0.064 | 0.415±0.029 | ** | NS | 8.296 | 0.002 |
| Citrate | 0.042±0.004 | 0.011±0.009 | 0.042±0.009 | NS | *** | 41.303 | <0.001 |
| Creatine | 0.293±0.074 | 0.672±0.133 | 0.739±0.255 | *** | NS | 13.867 | <0.001 |
| Choline | 0.090±0.022 | 0.161±0.016 | 0.129±0.034 | ** | * | 15.123 | <0.001 |
| PC* | 0.298±0.062 | 0.460±0.033 | 0.288±0.046 | NS | *** | 32.325 | <0.001 |
| GPC* | 2.820±0.297 | 1.382±0.094 | 1.652±0.116 | *** | ** | 124.912 | <0.001 |
| Taurine | 1.976±0.145 | 0.723±0.139 | 1.144±0.205 | *** | *** | 107.986 | <0.001 |
| Glycine | 0.388±0.061 | 0.505±0.106 | 0.414±0.077 | NS | * | 4.084 | 0.033 |
| Glycerol | 0.081±0.004 | 0.088±0.011 | 0.074±0.009 | NS | ** | 5.516 | 0.016 |
| Threonine | 0.217±0.030 | 0.317±0.070 | 0.247±0.033 | NS | ** | 8.398 | 0.002 |
| Glucose | 2.507±0.249 | 1.091±0.170 | 1.953±0.345 | ** | *** | 54.696 | <0.001 |
| Fumarate | 0.002±0.001 | 0.004±0.000 | 0.003±0.001 | * | ** | 15.829 | <0.001 |
| Tyrosine | 0.057±0.005 | 0.068±0.012 | 0.071±0.019 | NS | NS | 1.987 | 0.167 |
| Phenylalanine | 0.021±0.005 | 0.107±0.027 | 0.036±0.004 | NS | *** | 59.267 | <0.001 |
| Histidine | 0.037±0.005 | 0.033±0.004 | 0.036±0.005 | NS | NS | 1.509 | 0.245 |
| Formate | 0.022±0.002 | 0.037±0.007 | 0.024±0.005 | NS | *** | 19.357 | <0.001 |

*P<0.05，**P<0.01，***P<0.001。 *: 3-HB, 3-Hydroxybutyrate; PC, O-phosphocholine; GPC, sn-Glycero-3-phosphocholine.

**Table S3** comparison of metabolite levels among the control group, 8-week ACLT group, and 8-week sham group

| **Metabolite** | **Mean ± SD** | | | **Multiple comparisons**  **test** | | **One-way**  **ANOVA** | |
| --- | --- | --- | --- | --- | --- | --- | --- |
|  | **control** | **8w sham** | **8w ACLT** | **control**  **vs.**  **8wACLT** | **8wsham**  **vs.**  **8wACLT** | **F** | **P** |
| Valine | 0.222±0.018 | 0.289±0.038 | 0.287±0.019 | ** | NS | 13.178 | <0.001 |
| Isoleucine | 0.146±0.010 | 0.176±0.020 | 0.171±0.011 | ** | NS | 8.022 | 0.003 |
| Leucine | 0.125±0.008 | 0.137±0.014 | 0.126±0.008 | NS | NS | 2.830 | 0.085 |
| LDL/VLDL | 5.751±0.565 | 5.958±0.302 | 3.899±1.587 | ** | ** | 9.903 | 0.001 |
| 3-HB * | 0.273±0.063 | 0.175±0.047 | 0.229±0.025 | NS | NS | 7.645 | 0.004 |
| Lactate | 2.758±0.327 | 2.491±0.340 | 2.592±0.135 | NS | NS | 1.567 | 0.236 |
| Alanine | 1.161±0.195 | 0.920±0.065 | 1.010±0.131 | NS | NS | 5.730 | 0.012 |
| Lysine | 0.293±0.019 | 0.428±0.049 | 0.413±0.063 | *** | NS | 18.157 | <0.001 |
| Acetate | 0.189±0.022 | 0.095±0.022 | 0.131±0.045 | ** | * | 18.198 | <0.001 |
| Arginine | 0.045±0.008 | 0.057±0.008 | 0.063±0.015 | ** | NS | 5.457 | 0.014 |
| Pyruvate | 0.206±0.029 | 0.203±0.012 | 0.193±0.035 | NS | NS | 0.413 | 0.668 |
| Succinate | 0.040±0.010 | 0.044±0.016 | 0.117±0.097 | * | * | 4.441 | 0.027 |
| Glutamine | 0.533±0.111 | 0.375±0.029 | 0.364±0.041 | *** | NS | 12.748 | <0.001 |
| Citrate | 0.042±0.004 | 0.035±0.004 | 0.029±0.002 | *** | ** | 22.343 | <0.001 |
| Creatine | 0.293±0.074 | 0.340±0.062 | 0.326±0.089 | NS | NS | 0.784 | 0.472 |
| Choline | 0.090±0.022 | 0.142±0.030 | 0.133±0.013 | ** | NS | 10.183 | 0.001 |
| PC* | 0.298±0.062 | 0.222±0.027 | 0.194±0.037 | *** | NS | 10.125 | 0.001 |
| GPC* | 2.820±0.297 | 1.898±0.187 | 1.335±0.141 | *** | *** | 76.208 | <0.001 |
| Taurine | 1.976±0.145 | 0.707±0.133 | 0.651±0.084 | *** | NS | 247.348 | <0.001 |
| Glycine | 0.388±0.061 | 0.264±0.019 | 0.285±0.053 | ** | NS | 14.560 | <0.001 |
| Glycerol | 0.081±0.004 | 0.068±0.010 | 0.065±0.002 | ** | NS | 10.568 | 0.001 |
| Threonine | 0.217±0.030 | 0.170±0.013 | 0.160±0.024 | *** | NS | 12.063 | <0.001 |
| Glucose | 2.507±0.249 | 2.039±0.229 | 3.123±1.075 | NS | ** | 5.552 | 0.013 |
| Fumarate | 0.002±0.001 | 0.002±0.001 | 0.004±0.002 | * | * | 3.245 | 0.063 |
| Tyrosine | 0.057±0.005 | 0.048±0.006 | 0.058±0.010 | NS | * | 4.689 | 0.023 |
| Phenylalanine | 0.021±0.005 | 0.029±0.003 | 0.039±0.003 | *** | *** | 40.858 | <0.001 |
| Histidine | 0.037±0.005 | 0.024±0.003 | 0.025±0.004 | *** | NS | 25.892 | <0.001 |
| Formate | 0.022±0.002 | 0.014±0.002 | 0.012±0.003 | *** | NS | 42.149 | <0.001 |

*P<0.05，**P<0.01，***P<0.001。*: 3-HB, 3-Hydroxybutyrate; PC, O-phosphocholine; GPC, sn-Glycero-3-phosphocholine.

**Table S4** comparison of metabolite levels among the control group, 12-week ACLT group, and 12-week sham group

| **Metabolite** | **Mean ± SD** | | | **Multiple comparisons**  **test** | | **One-way**  **ANOVA** | |
| --- | --- | --- | --- | --- | --- | --- | --- |
|  | **control** | **12w sham** | **12w ACLT** | **control**  **vs.**  **12wACLT** | **12wsham**  **vs.**  **12wACLT** | **F** | **P** |
| Valine | 0.222±0.018 | 0.384±0.113 | 0.335±0.051 | ** | NS | 9.235 | 0.001 |
| Isoleucine | 0.146±0.010 | 0.237±0.064 | 0.212±0.041 | * | NS | 8.066 | 0.003 |
| Leucine | 0.125±0.008 | 0.166±0.044 | 0.147±0.024 | NS | NS | 3.612 | 0.046 |
| LDL/VLDL | 5.751±0.565 | 4.107±1.255 | 5.178±0.868 | NS | * | 5.803 | 0.010 |
| 3-HB * | 0.273±0.063 | 0.472±0.195 | 0.427±0.202 | NS | NS | 2.793 | 0.085 |
| Lactate | 2.758±0.327 | 2.705±0.797 | 2.688±0.624 | NS | NS | 0.025 | 0.975 |
| Alanine | 1.161±0.195 | 1.079±0.183 | 1.030±0.213 | NS | NS | 0.830 | 0.450 |
| Lysine | 0.293±0.019 | 0.481±0.130 | 0.432±0.095 | * | NS | 7.598 | 0..004 |
| Acetate | 0.189±0.022 | 0.097±0.023 | 0.082±0.014 | *** | NS | 60.163 | <0.001 |
| Arginine | 0.045±0.008 | 0.073±0.025 | 0.060±0.014 | NS | NS | 4.768 | 0.020 |
| Pyruvate | 0.206±0.029 | 0.205±0.082 | 0.210±0.054 | NS | NS | 0.018 | 0.982 |
| Succinate | 0.040±0.010 | 0.060±0.043 | 0.046±0.009 | NS | NS | 1.098 | 0.353 |
| Glutamine | 0.533±0.111 | 0.467±0.114 | 0.435±0.074 | NS | NS | 1.829 | 0.186 |
| Citrate | 0.042±0.004 | 0.037±0.012 | 0.034±0.008 | NS | NS | 1.697 | 0.209 |
| Creatine | 0.293±0.074 | 0.774±0.221 | 0.623±0.190 | ** | NS | 14.259 | <0.001 |
| Choline | 0.090±0.022 | 0.234±0.088 | 0.192±0.054 | ** | NS | 10.446 | 0.001 |
| PC* | 0.298±0.062 | 0.261±0.022 | 0.283±0.043 | NS | NS | 1.326 | 0.288 |
| GPC* | 2.820±0.297 | 1.628±0.143 | 1.696±0.277 | *** | NS | 54.057 | <0.001 |
| Taurine | 1.976±0.145 | 0.782±0.179 | 0.731±0.224 | *** | NS | 103.629 | <0.001 |
| Glycine | 0.388±0.061 | 0.428±0.093 | 0.352±0.084 | NS | NS | 1.759 | 0.198 |
| Glycerol | 0.081±0.004 | 0.093±0.010 | 0.080±0.010 | NS | ** | 5.755 | 0.011 |
| Threonine | 0.217±0.030 | 0.246±0.040 | 0.222±0.033 | NS | NS | 1.551 | 0.236 |
| Glucose | 2.507±0.249 | 2.478±0.684 | 2.044±0.294 | NS | NS | 2.462 | 0.111 |
| Fumarate | 0.002±0.001 | 0.003±0.001 | 0.003±0.001 | NS | NS | 1.813 | 0.189 |
| Tyrosine | 0.057±0.005 | 0.062±0.014 | 0.056±0.010 | NS | NS | 0.723 | 0.498 |
| Phenylalanine | 0.021±0.005 | 0.046±0.007 | 0.047±0.009 | *** | NS | 28.267 | <0.001 |
| Histidine | 0.037±0.005 | 0.031±0.005 | 0.027±0.005 | ** | NS | 6.805 | 0.006 |
| Formate | 0.022±0.002 | 0.018±0.005 | 0.018±0.004 | * | NS | 2.832 | 0.083 |

*P<0.05，**P<0.01，***P<0.001。*: 3-HB, 3-Hydroxybutyrate; PC, O-phosphocholine; GPC, sn-Glycero-3-phosphocholine.

**Table S5** Significantly altered metabolic pathways identified in different postoperative stages of rat serum

| **Pathway** | **4w sham**  **vs.**  **4w ACLT** | **8w sham**  **vs.**  **8w ACLT** | **control**  **vs.**  **4w ACLT** | **control**  **vs.**  **8w ACLT** | **control**  **vs.**  **12w ACLT** |
| --- | --- | --- | --- | --- | --- |
| 1. Phenylalanine, tyrosine and tryptophan biosynthesis | √ | √ | √ | √ | √ |
| 2. Phenylalanine metabolism | √ | √ | √ | √ | √ |
| 3. Pyruvate metabolism | √ | √ | √ | √ | √ |
| 4. Taurine and hypotaurine metabolism | √ | × | √ | √ | √ |
| 5. Starch and sucrose metabolism | √ | √ | × | √ | × |
| 6. Glycine, serine and threonine metabolism | √ | × | √ | √ | √ |
| 7. Histidine metabolism | √ | √ | × | × | × |

**Figure legends**

**Fig. S1** The weight change trend of rats (A-C).

**Fig. S2** Typical 1D NMR spectrum from rat serum sample. The water region (4.8-5.1 ppm) was removed. The region of 5.1-9.0 ppm (in the dashed box) was magnified 10 times compared with the corresponding region of 0.8-4.7 ppm for clarity. Abbreviations: 3-HB, 3-Hydroxybutyrate; PC, O-phosphocholine; GPC, sn-Glycero-3-phosphocholine.

**Fig. S3** Typical 2D 1H-13C HSQC spectrum of aqueous extracts.

**Fig. S4** Region ppm of a typical 2D 1H-1H TOCSY spectrum of aqueous extracts (A-B).

**Fig. S5** VIP score-ranking plots of significant metabolites identified from the PLS-DA models. (A) control group vs. 4-week ACLT group; (B) 4-week sham group vs. 4-week ACLT group; (C) control group vs. 8-week ACLT group; (D) 8-week sham group vs. 8-week ACLT group; (E) control group vs. 12-week ACLT group.
